# Organoid Ethical Typology: varieties of three‐dimensional stem cell constructs and the many issues they raise in bioethics

**DOI:** 10.1111/boc.202400093

**Published:** 2025-01-24

**Authors:** Maxence Gaillard, Charles H. Pence, Mylène Botbol‐Baum

**Affiliations:** ^1^ Centre for Medical Ethics Oslo (Norway) & Institut supérieur de philosophie University of Oslo, UCLouvain Louvain‐la‐Neuve Belgium; ^2^ Institut supérieur de philosophie UCLouvain Louvain‐la‐Neuve Belgium

**Keywords:** bioethics, biotechnology, ethics of science and medicine, organoids, stem cells

## Abstract

The advancement of and prospects for stem cell research raise a number of specific ethical issues. While navigating the ethical landscape of stem cell research is often challenging for biology researchers and biotechnology innovators, it is also difficult for the public and other persons of concern (from ethicists to policy‐makers) to grasp the technicalities of a burgeoning field that develops in many directions. Organoids are one of these new biotechnological constructs that are currently eliciting a rich debate in bioethics. In this guide, we argue that different types of organoids have different emerging properties with different ethical implications. Going from general properties to particular ones, we propose a typology of organoid technology and other associated biotechnology from a philosophical and ethical perspective. We point to relevant ethical issues and try to convey the sense of uncertainty peculiar to ongoing research and emerging technological objects.

## Graphical abstract

Biotechnological stem cell constructs, especially organoids, are eliciting a rich debate in bioethics. However, different types of organoids have different emerging properties with different ethical implications. Confusing existing issues is a source of misunderstanding and miscommunication that can prevent the ethical flourishing of a promising research field. This article proposes a typology of organoid from a bioethics perspective to help scientists navigate the ethical landscape.

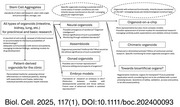

**Abbreviations**
3D: three‐dimensional
eSC: embryonic stem cells
iPSC: induced pluripotent stem cells
ISSCR: International Society for Stem Cell Research
OOC: organoid‐on‐chip
SC: stem cell
TRL: technology readiness level

## INTRODUCTION: PROPOSAL FOR A TYPOLOGY

“Organoid” refers intuitively to an entity that is similar to an organ or has the shape of an organ—an asteroid is a celestial body that looks like a planet or a star (*aster*) but is actually not. Although the term was used in biology before the 20th century (Davies, 2018), it has been given a more specific meaning recently. In the context of stem cell research and new cell culture techniques, organoids are generally defined as three‐dimensional stem cell cultures that replicate, in some ways, the structure and functions of organs. There are now many definitions of an organoid available in the literature, such as: “a 3D structure grown from stem cells and consisting of organ‐specific cell types that self‐organizes through cell sorting and spatially restricted lineage commitment” (Clevers, 2016) or “a stem cell‐derived complex 3D structure with the architecture and functionality of a normal organ” (Mummery et al., 2021). A detailed analysis could uncover subtle semantic distinctions between these definitions. Still, they all revolve around the same points: a three‐dimensional cell culture that comes from stem cells that differentiate and self‐organize, up to the point that they reproduce, at a small scale, some properties (functional and structural) of an actual biological system.

This new biotechnology emerged in the 2010s, as the term “organoid” was forged in its current sense in an article from Hans Clevers’ laboratory (Sato et al., 2009). Although it is debatable whether 3D stem cell culture was a brand‐new technique at the time (Simian & Bissell, 2017), the term has since become a widely adopted keyword for a technology covering many organs and physiological systems (Shoji et al., 2023). Claiming that organoids are new entities (in the large family of contemporary biotechnology) amounts to assuming that they cannot be genuinely reduced to any simpler in vitro cell culture.

According to this perspective, there would be something more in an organoid than in a traditional cell culture. Indeed, organoids are recognized by most actors of the field as a new biotechnology because they have some properties that other cell cultures do not have—at least, this is what is assumed when their unique ability to mimic organs in vitro is put at the forefront. In other words, organoids are different from simple cell cultures because they have some emerging properties that are added to the properties of the regular cell culture.

Our main hypothesis here is that some of these emerging biological properties are of significance for ethical assessment as well. Of course, ethical provisions with regard to research and the clinic can be both general and specific to a concrete object of concern. Every domain of science and technology raises specific ethical issues when it comes to its own prospects: we will neither have the same risks and benefits, nor the same monitoring procedures, for research aiming at—for instance—building a model of climate, a model of a black hole, a model of a virus, a model of the pancreas, a model of an embryo, and so on. Whether and how exactly the emerging biological properties of a new entity of interest are of ethical significance and deserve specific consideration is still an open question.

Biomedical research has been working on cell cultures for decades, admittedly with its fair share of unethical practices (see the Henrietta Lacks affair), but there are now multiple ethical frameworks and a body of standards to rely on. Some of the questions raised by organoid research are classic issues when biomedical research deals with human biomaterials, such as informed consent, biobank management, or commodification of cell lines. But—and this is our focus here—there might be also new, specific issues raised by progress in the field. The interested reader can refer to existing reviews of ethical issues pertaining to organoid technology for others’ attempts to answer these questions (Mollaki, 2021; Barnhart & Dierickx, 2022; de Jongh et al., 2023). Our approach here is to depart from the insight that some of the distinct entities falling under the umbrella of organoids and related technologies might not be ethically significant in the same way. What we want to achieve here in a succinct format, then, is an overview of ethical issues that is based on the distinctive, emergent properties of the entities of concern.

This is why we propose here a typology of different entities related to organoid research. A typology is a more than a list: it provides a classification, based on explicit criteria, of types of entities that fall within certain categories. A typology differs from a nomenclature, which is mostly about imposing guidelines for naming, or labelling, different entities. There are many calls for building robust nomenclatures with regard to, for example, hepatic organoids (Marsee et al., 2021), embryo models (Matthews et al. 2021), or nervous system organoids (Pașca et al., 2022). As important as it is for scientific standards and the communication of science, nomenclatures and the issue of how one should label various biotechnological entities are only a small part of research ethics. Rather than a normative framework, our typology is intended to be a tool or a guide to navigate the field of ethical issues raised by these entities.

In every classification, there is inherent choice, as one could classify the same collection of entities on different grounds by using different classification criteria. For instance, the source of the stem cell (e.g., human embryonic SC vs. induced pluripotent SC) is probably a good criterion to delineate ethical issues related to the “in vitro embryo debate” on one hand and the collection of cells from living donors on the other (Hyun, 2013). However, this will be true only to a certain point, after which there will remain common issues. For instance, if one wants to put scrutiny on the (even remote) possibility of obtaining brain organoids that are conscious, whether these organoids are made of eSC or iPSc is not likely to become the crux of the matter. Considering another classification, the International Society for Stem Cell Research proposes distinct categories for research review and oversight (ISSCR, 2021). A research project will then fall within a given category or another based on the degree of ethical sensitivity of the research. For instance, research listed in category 2 is advised to be reviewed by a dedicated committee or to undergo a specialized oversight process, and the guidelines list the kinds of research that fall within this category (ISSCR, 2021). This is a very pragmatic tool for researchers, but at the same time very normative. As research moves forward, some objects might move from one category to another. Some models that were “exempt from further review” in a previous stage of technique would become “in need of a specific review” after they reach a certain degree of complexity and functionality. While guidelines can deal with this kind of situation with recurrent updates, the typology that we propose here is intended to foster open‐ended reflection on different entities of concern that are easy to identify. As science advances and better models are developed, these entities will evolve and new properties may emerge: we do not propose a definitive framework for ethical thinking but only indications for addressing the right questions in the right place.

**FIGURE 1:**
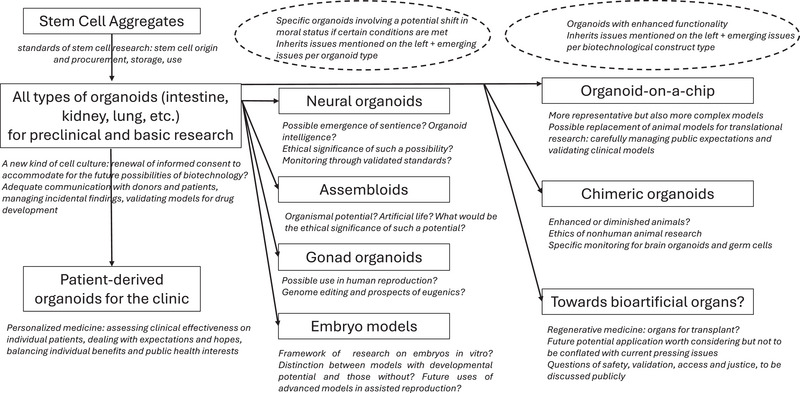
Typology of organoids and main questions.

We start from the idea that organoid technology is built on stem cell research (a field with its own ethical concerns) and that more complex entities are progressively emerging as stem cell cultures develop in specific directions. As a consequence, the typology looks like a flowchart, with branches for entities that deserve specific ethical consideration (see Figure 1). There are several constraints that shape this final graph. Research objects are evolving entities: Liver or brain organoids in 2022 are not the same objects as liver or brain organoids will be in 2026. This is the reason why the proposed typology does not aim simply to follow the categories that might be drawn in a purely scientific or biological context. Put differently, some properties that a biology researcher would deem important in describing a given entity and which are of interest for biology are not necessarily ethically relevant, that is, they do not turn this new biotechnology into a brand‐new entity of concern for ethics. Overlooking the most subtle and state‐of‐the‐art scientific details and keeping only the aspects that are most significant from the viewpoint of ethics and society, we have tried to find a balance between general provisions that would apply indifferently to all cell cultures and the impossible task of analyzing the specific context of every laboratory. If a distinction is found within our typology, we believe that it is a distinction that makes a genuine ethical difference.

A last point of attention for reading Figure 1 is that each branch inherits issues discussed at the previous stage. As entities grow more complex, new points of concern do not replace former ones, but instead, these concerns accumulate. For instance, the fact that brain organoids raise new debates related to the potential emergence of consciousness in vitro is a specific point of concern for brain organoids, but donor consent and biobanking management are still to be taken into consideration for all stem cell constructs (that is, including brain organoids).

Note that all the biotechnological entities discussed below are not at the same level of maturity. Technologists and research funding organizations typically refer to the “technology readiness level” (TRL) to position an innovation along a scale of development, although the identification of the TRL for a given technology often comes with many uncertainties and approximations. Under this framework, different applications and different types of organoids are not at the same TRL. For instance, patient‐derived organoids are already the topic of 100+ clinical trials while bioartificial organs remain mostly conceptual. Brain neural organoids are still very crude research tools compared to actual mammal brains. As a consequence, some of the questions mentioned here are anticipatory in the sense that the technology is not yet at the point where the most pressing issues will arise. It is nonetheless important to address these issues beforehand, while being aware of the TRL of each envisioned application to avoid hype and misleading the public in this respect (Pichl, 2023).

## FROM STEM CELLS TO ORGANOIDS

Organoid research can be considered one of the latest developments of stem cell research: research on and with human stem cells that has seen continuous progress since James Thomson produced the first human embryonic stem cell line (Thomson et al. 1998). Since then, there have been debates on stem cell procurement and the possibility of new treatments through cell therapy and regenerative medicine. In other words, stem cell research has been a controversial field since its inception. In the US, the topic has been at the forefront of political debates at the turn of the century, with very opposing views from conservatives and liberals and different legislation at the federal and state levels (Scott, 2005). In Europe, the diversity of cultures was an obstacle to defining a common position among EU members, and today's international regulatory landscape is still difficult for researchers to navigate. Sociologist Sheila Jasanoff (2007), in a comparison of the relation of bioethics to politics across countries, goes on to say that the stem cell debate even played a role in the institutionalization of bioethics as such.

If organoid research is an expansion of stem cell research, then organoid ethics should be seen as an expansion of stem cell ethics. Organoids being made from stem cells (even if stem cells might not be present in organoids any longer, as they would have differentiated), stem cell ethics is a minimal requirement for the ethical development of organoids. Several aspects drawn from this field can be mentioned, such as the procurement of the cells, the responsible conduct of research, and management of the expectations of donors and the public.

Stem cell procurement raises different issues depending on the source of the cells and the type of biomaterial used. Most of the debate has so far focused on the collection of embryos. The use of excess in vitro fecundation embryos for research purposes has raised a public discussion about the moral status of the embryo and whether researchers should be allowed to use them as a source of material. Oocyte procurement is also a controversial practice, giving rise to issues with egg donor compensation, which can be seen as either exploitation or unfair inducement. Stem cells collected from consenting donors, for instance, to be reprogrammed as induced pluripotent stem cells, raise the issue of donors’ information and consent. Although procedures for informed consent are already in force, organoids might force us to renew these procedures and re‐consider their meaning (see below).

This calls for an important distinction between ethical issues related to the procurement of stem cells and the development of stem cell‐derived entities in the laboratory. Bioethicist Insoo Hyun (2013) insists on maintaining the distinction between the “embryo debate” and the “stem cell controversy.” The embryo debate focuses on the fate of embryos and asks whether researchers should be allowed to work with material from embryos which, from certain philosophical standpoints, are seen as potential persons. However, the actual scope of the stem cell debate is much larger, and the bioethical discussion on the future of stem cell research has already moved forward. According to Hyun, stem cell research interrogates our ability to control life, master biological development, and play with its plasticity. Ultimately, our power over nature and our attitudes toward aging and disease are at stake, through the expected contributions of stem cell research and biotechnologies derived from stem cells to regenerative medicine and human enhancement. Therefore, stem cell research ethics deals not only with the issue of whether scientists should use stem cells from embryos or not, but also questions a whole range of possibilities and procedures for producing laboratory entities derived from stem cells.

The first, and, in a sense, most primitive, kind of biotechnological entity obtained when culturing stem cells in three dimensions, is what we could call STEM CELL AGGREGATES, which appear after a period of in vitro cultivation. Stem cell aggregates include entities such as teratomas (human stem cells grown as tumors in mice), embryoid bodies (small spheres including the three germ layers), and spheroids. These entities include stem cells that are differentiating or have differentiated up to a certain extent, as they can contain different tissue types (for embryoid bodies and teratomas), but the cell cultures do not exhibit a complex spatial layout or an organization that is typical of any actual organ or stage of development. These entities have been present in the laboratory for decades: teratomas as assays for stemness and embryoid bodies as sources of stem cells for all ranges of studies, for example, for neural stem cells to grow brain organoids. The culture of stem cell aggregates raises ethical and practical issues on its own, such as quality control and good manufacturing practice (cell culture requires delicate handling and precise documentation), before their potential storage or commodification as laboratory products or medical applications. We argue nonetheless that stem cell aggregates, as precursors of organoid technology, are not seen at this stage as a source of specific ethical issues in the sense that we will elaborate below.

Beyond simple aggregates or spheres, researchers usually refer to ORGANOIDS when some basic level of architecture and functionality emerges from the three‐dimensional stem cell culture. The prototypical notion of organoid would refer to a model of an organ of the body (liver, gut, or brain…) that has, in some sense, “the architecture and functionality of a normal organ” (Mummery et al., 2021).

In that sense, ethical issues already emerge from the fact that the biotechnological construct that researchers are aiming at will look like an organ. One of the first issues pertains to cell donation and informed consent of donors. Informed consent of donors is a prerequisite for conducting research on donated biomaterial. For cell lines that already exist or material already collected, even if the donor has already consented to the use of their material in broad terms, consent was probably given without the donor and researchers considering the very possibility of replicating organs in vitro. How can one ensure that consent is actually informed in such a context (Solbakk et al., 2009; Boers & Bredenoord, 2018)? Does uncertainty about the future possibilities of biotechnology nullify the right of researchers to build new biotechnological entities from material already collected, or to conduct a battery of tests such as genomic sequencing? What is tolerated for existing cell lines cell lines entrenched in daily lab practice might not be for newly collected material. A reflection upon the management of consent through time has been launched: is broad initial consent sufficient, or should consent be demanded again—according to a “dynamic consent” framework—when substantively new progress in cell culture is made (Isasi et al., 2024)?

Informing donors that their own cells could be grown into organoids can be challenging. Pitfalls include miscommunication on the nature of organoids (which can be described as “mini‐organs” in vitro) and therapeutic misconceptions (when patients expect clinical benefits at the individual level, while most research is basic or preclinical research that contributes to the advancement of knowledge but not to personalized medicine). Sufficient information should be provided concerning the future uses of derived products, such as their circulation among laboratories and countries, their integration in other biotechnological constructs, and their potential commercialization. Donors should be aware that giving to a biobank is a kind of future‐oriented commitment that might not be reversible. The question of relations with the donor also includes the issues of anonymization of data and the treatment of potential incidental findings. For instance, if organoids as models of organs make anonymization more difficult or incidental findings more frequent and accurate, the degree of protection of donors will have to be upgraded.

Supplementary considerations might apply for specific applications, such as PATIENT‐DERIVED ORGANOIDS for precision medicine. Tumoroids, models of tumors usually made from tumor resection or biopsy, are one example of a technology that can be used either to understand cancer development from a basic research perspective and to test the efficacy of drugs in preclinical research, or to predict the best treatment for a given patient according to the paradigm of personalized medicine. Properly speaking, tumors are not organs, and the architecture and functionality, so to speak, of tumoroids are still rather simple compared with models of organs of the body. From an ethical viewpoint, tumoroids as tools for basic research on the mechanisms of cancer and for drug testing in preclinical research are not different from other entities (tumor‐derived spheroids, cancer cell cultures, etc.). Yet patient‐derived tumor organoids might be considered to pose distinctive ethical questions in the context of personalized medicine. When cancer cells are taken from the patient's own tumor and grown in vitro as a personalized model of the tumor, it is expected that tests performed on this individual tumor model will benefit the donor. Will functional precision medicine really make a leap forward thanks to patient‐derived organoids (Vogt et al., 2025)? An important point to consider is that donors should neither be confused about the goal of their donation (especially that they do not expect personal benefits when they donate for basic research), nor overconfident regarding the prospects of experimental personalized medicine. Experimental personalized medicine forces us to navigate the grey zone between care and research: How do we assess the clinical efficiency of procedures for individual patients? How do we identify the relevant regulatory framework for administering drugs predicted by an assay that is not routine clinical practice (e.g., compassionate use)? Finally, the costs and benefits of personalized medicine for society as a whole should be weighed, and its impact on the healthcare system discussed.

## SHIFTING MORAL STATUS?

NEURAL ORGANOIDS are models of the early stages of development of the nervous system or some parts of the early brain, built from the 3D culture of neural stem cells. For animals, the nervous system is key in the emergence of mental phenomena such as sentience, the ability to feel pleasure, discomfort or pain. Consciousness, as the emergence of something like subjective experience, is also dependent on the functioning of the nervous system. If a complex neural organoid were to acquire some properties such as sentience or consciousness, then it would qualify for a certain moral status, even as a research object (Lavazza & Massimini, 2018; Greely, 2021). The prospects of intentionally building artificial computing systems based on “organoid intelligence” reinforce this concern (Smirnova et al., 2023). Potentially sentient organoids ought to be treated on a par with other sentient entities used in the lab (e.g., animal models). Should we refuse the development of such entities or accept them only under certain conditions (e.g., if the suffering imposed on them is negligible compared to the benefits that actual human beings will gain from this research)? Or, on the contrary, is the emergence of such functions possible at all from models in vitro? How should we assess this putative emergence? Not all neural organoids will have this potential, which will likely depend on reaching a critical size, a complex architecture and degree of maturation, as well as establishing a connection with their environment (inputs and outputs). Even if there is a long way to go before neural organoid culture reaches a level of development that should raise actual concerns, questions about the implementation of procedures for assessing their potential sentience as well as standards to ensure non‐sentience or minimal sentience can already be debated (Birch, 2024).

ASSEMBLOIDS are combinations of organoids. As several functional organ‐like structures are grown separately and then cultured together, assembloids might be able to develop an entire system with interacting components. From an ethical viewpoint, there are two distinctive concerns that arise here. The first one pertains to assembloids involving neural organoids. Such a construct might be seen as “enhancing” brain organoids by embodying them (e.g., connecting neural organoids with other organ models such as sensory organs or muscle), and embodiment could pave the way to the development of sentience. The second concern is related to the organismal potential of biotechnological constructs. While the purpose of assembloid technology is to build more realistic physiological systems that include interaction between body parts, we need to consider the boundaries of how realistic we want these models to become. According to bioethicist Henry Greely (2021), this is an ethical dilemma inherent to surrogate models: Researchers build and use models because they do not want to or are not allowed to work on the real thing. However, when the model gets closer to the real thing it is intended to model, then the very same ethical issues reemerge. This is already the case with animal models—animal experimentation is justified because we do not want to experiment on human beings, but the animals most similar to humans are also those that raise the most ethical reservations. However, the dilemma could soon be an issue for biotechnology as well. Looking at models of organs separately does not incline us to worry about their moral status, as heart cells beating synchronously in a dish does certainly not qualify for life and the contraction of muscle tissue in vitro does not equate to voluntary movement. But what if we connect all these biotechnological constructs in a complex and interactive system? How many organs does it take to make an organism? Can research arrive at systems that self‐maintain or self‐regulate their internal environment? The organismal potential of a stem cell‐based model would be a new, emerging property, the moral significance of which would have to be assessed, possibly following the lines of the debate on synthetic biology and artificial life.

GONAD ORGANOIDS are models of the organs of reproduction. Even if, at this stage, the primary purpose of basic research is the understanding of infertility and its mechanisms, the perspective of developing gametes in vitro is a long‐standing one in assisted reproductive technology (with, e.g., the production of gametes directly from pluripotent stem cells). Along with other gametogenesis technologies, gonad organoids raise the question of their use for reproduction and its potential impact on the future of humankind (Mathews et al., 2009; Ishii et al., 2013; Greely, 2018). While assisted reproduction techniques, including in vitro fertilization, are largely considered to answer a legitimate demand, the societal acceptance of manipulation and selection of heritable traits is much more debated, as it might lead to eugenics. For instance, genome editing to modify the human lineage is currently banned according to most jurisdictions and according to most ethical guidelines. The use of organoids for reproduction would amount to a shift in their status, from models to actual components of life. Such a shift should be carefully assessed, not only in terms of safety of the procedure, but also in terms of its desirability and prospects for humankind. In that sense, it joins concerns raised by stem cell‐based embryo models if they were turned into something like synthetic embryos (see below).

EMBRYO MODELS are stem cell‐derived entities that build on organoid technology to replicate in vitro the early stages of embryo development. Here, one should carefully distinguish between research ethics and reproductive ethics. With regard to research ethics, what is the moral and legal status of these bioengineered entities partially akin to embryos? This is a pressing issue for laboratory researchers and lawmakers today, with many ongoing initiatives discussing the topic worldwide (Fabbri et al., 2023). Embryo models are not fertilized embryos, hence in most jurisdictions the laws governing embryo research do not apply to these entities (for instance, the 14‐day rule, which claims that embryos shall not be cultivated in vitro more than 14 days post‐fertilization). However, as technology improves, the boundaries between models and actual embryos will blur. Should limits be imposed on the in vitro culture of embryo models and should dedicated oversight committees supervise this work, comparable to the limits and oversight committees of embryo research? The ISSCR (2021) popularized a distinction between (i) integrated embryo models, containing all the relevant embryonic and extra‐embryonic structures, that would have, under certain conditions, the potential to become a fetus if cultured for additional time and transplanted in vivo and (ii) non‐integrated models that have no such potential. It is often taken for granted that the first kind of model deserves specific attention and high ethical consideration while the second one should be considered less problematic. It is, however, a challenge to assess the developmental potential of these entities and to infer ethical implications. The challenge of assessing potentiality and the variety of models developed in research settings do not necessarily make the distinction between integrated and non‐integrated models the best rule of thumb available for law. Only careful monitoring of the developmental potential of the models can guide ethical decision‐making and ensure that stem cell‐based embryos with the same potential as natural embryos should be treated in the same manner (Rivron et al., 2023).

If the possibility of obtaining viable embryos from stem cells were realized in the future, supplementary but distinct questions would be raised in reproductive ethics. In the current state of the technology, implanting a stem cell‐derived embryo model would be both unsafe and lacking any scientific rationale. Yet, if we could create embryos from stem cells, would we do so, and what would be the consequences for humankind? In search of ethical guidance, one could draw an analogy with cloning. The prohibition of cloning (as the creation of a human being genetically identical to another one) as well as the current ban on heritable genome editing makes the acceptance of such synthetic embryos unlikely. However, should we consider it a temporary ban—because the technology comes with too many uncertainties today and would infringe on the basic safety of putative research participants—that might be lifted one day? This is, again, a societal issue that should be debated at large (Howard et al., 2018).

## ENHANCED ORGANOIDS

Beyond organoids as entities self‐organizing in a dish, the advances in microfluidics and artificial scaffold technology can also bring stem cell constructs to another level by building ORGANOIDS‐ON‐CHIP (OOC). Although there is a range of microphysiological systems that can be regrouped under this label, the artificial structure allows, in general, the reproduction and control of the chemical environment and mechanical constraints on the target organ, enhancing the functionality of the organoid model. Issues in good manufacturing practice and standardization might be slightly different in the sense that OOC technology includes some non‐biological material (the “chip”), but OOC technology is used in basic and preclinical research in a manner comparable to organoid technology and is thus likely to raise similar ethical issues. When combined, OOCs of different bodily organs may exchange fluids in a setup that makes blood‐like circulation and body physiology possible. These “multi‐organ body‐on‐chip systems” (Ingber, 2022), which offer more and more realistic testing conditions for the absorption and distribution of drugs in the body, are likely to raise issues similar to assembloids—developing properties akin to an organism or a subsystem of it. Another interesting emerging property is the prospect of using this technology to bridge the gap between human cell‐based but unrealistic cell cultures and animal models that are physiologically relevant but not from the human species, and thus provide an opportunity to replace and reduce animal models in translational research (Stresser et al., 2023). Although the prospect is exciting from an ethical point of view, hype should be avoided because there is still a long way to go, first to collect evidence on the safety and clinical effectiveness of these systems in order to validate these new alternative methods, and then to change relevant scientific and regulatory practices (Lohse, 2021).

Another biotechnological construct from stem cells that deserves attention in the context of organoid technology are CHIMERAS, organisms composed of cells from different genetic origins. The most ethically concerning chimeras are those mixing human cells with animal genetic material. The boundary between human beings and other animals is allegedly crucial when it comes to moral status and attribution of rights. It has been argued that the mere existence of chimeras, entities that could not be classified as either humans or animals, could challenge our moral categories (Robert & Baylis, 2003), although it is debatable whether local laboratory practice can have such major anthropological consequences (Hübner, 2018). If the chimeric models are produced by mixing animal and human stem cells at the embryo level or introducing human stem cells into animal embryos, researchers will obtain chimeric embryos. The properties of chimeric embryos (and the ensuing ethical issues) may vary according to the degree of chimerism, or the proportion of human cells in the mix. Another option for creating chimeras is the transfer of organoids derived from human stem cells into postnatal animal hosts. The ethical assessment of this practice should consider the species involved (there are specific concerns with chimeras involving non‐human primates given their proximity to the human species) and the organ grafted, with supplementary caution with regard to the organs of interest already mentioned, such as the brain and the reproductive tract. In the case of brain organoid grafts, the possible induction of behavioral and cognitive changes in the animal host should be assessed and monitored. In all cases, the ethics of research on nonhuman applies to research on chimeric animals, suggesting that the scientific rationale for such research and the welfare of animals are key (Johnston et al., 2022).

## TOWARDS BIOARTIFICIAL ORGANS?

The main applications of organoid technology today are models for developmental biology, disease modeling, drug screening and development. However, there is a vision latent in many discourses presenting organoids as “mini‐organs” and tools for regenerative medicine. On this hypothesis, organoids could reach an advanced stage of development, so that they look like, or present functional similarities with, actual bodily organs or parts of organs. As they are currently models of development limited in size (a few millimeters) and functionality, this vision assumes that organoids will be pushed to a much higher level. In the best technological scenario, they would contribute to BIOARTIFICIAL ORGANS as part of a strategy to supply organs for transplants.

While reiterating that organoids as models of development are not mini‐organs, it is nonetheless a good idea to allow for discussion of the future‐oriented vision of bioartificial organs. There is indeed a long tradition of attempts to develop artificial organs for clinical purposes (Duguet, 2021). Regenerative medicine faces many hurdles, from ethical issues when it comes to transplantation of organs obtained from dead or living donors, to technical limitations and risks of these procedures, including reactions of the immune system—notwithstanding the longstanding ethical issue that there are many more patients in need than organs made available for transplant. Through this lens, a technology that would provide safe and ethical organs, possibly from patients’ own stem cells, would represent a tremendous opportunity for regenerative medicine. Organoids would be one option in the toolbox of bioartificial organs for regenerative medicine, along with bioprinting, xenotransplantation, and so on. There are classic issues here, such as assessing the safety and efficacy of the surgical procedure, especially in pioneering cases (Xinaris, 2019). The regulatory status of the manufacturing process would have to find its place in a landscape of procedures, as organoids‐for‐transplant would be bioengineered entities, somewhere between natural organs collected from donors, cell therapies, and artificial organs (e.g., mechanical valves) or prostheses. As organoid research will contribute to the development of bioartificial organs, questions regarding their regulatory classification and a systematic comparison of the risks and benefits of different types of bioartificial and artificial organs would have to be discussed.

Furthermore, the prospects of developing bioartificial organs raises deeper issues related to the representation of the body and how engineered tissues are expected to integrate into the “lived body” (Derksen & Horstman, 2008) or impact the sense of personal identity (Hansson, 2005). Hype and media uptake are also a common concern for responsibly managing public expectations in regenerative medicine (Vermeulen, 2017). Any public strategy of investment in bioartificial organs will also have to address the legitimacy of their purpose—restoration or enhancement—as well as their cost and availability for patients who really need them.

## CONCLUDING REMARKS

The advancing development of biotechnology is likely to raise ethical issues, because the new biological properties that these entities take on will force us to look differently at them and at their possible uses. In this sense, entities developed in the lab not only inherit the properties of their precursors, but also can raise novel ethical concerns. We have sketched a typology of organoids with these concerns in mind, and connected this typology to the different ethical questions these entities raise. We came across three kinds of unresolved issues, each corresponding to a path towards their resolution:

‐ Traditional ethical issues that scale up because of the development of organoids as entities of a new kind. These imply, among other issues, questioning the significance of informed consent and possibly reconsidering best practices for the management of biomaterial in biobanking and research, ensuring that trust in science is maintained through respect for donors and appropriate communication about the potentiality and validity of models.
‐ The possible emergence of new properties of more serious ethical significance, such as sentience (neural organoids) and organismal potential (embryo models, assembloids). These properties might in some cases push us to reconsider the moral status of the entities of concern. The difficulty, in this case, lies in identifying the actual moral significance of the emerging properties and in defining validated procedures for monitoring their potential emergence in the lab. Current organoid models are still tools, and the priority should be put on tool development on a par with methods to assess these tools and to better know what they are capable of.
‐ The development of applications that will profoundly impact healthcare and society, and that need to be publicly debated as such. Such developments include the advancement of regenerative medicine (bioartificial organs) and personalized medicine (patient‐derived organoids), as well as new perspectives on human reproduction (embryo models, gonad organoids). As promising and fascinating as organoid technology can be, the most important point here is that all these distinct applications deserve to be delineated precisely, and their desirability discussed publicly. It is one thing to grow in vitro complex cellular models to understand developmental mechanisms, and another to build organs from stem cells. Even if the former might pave the way to the latter, social acceptance and relevance should not be assumed, and not all engineers’ dreams are predestined to come true.

## CONFLICT OF INTEREST STATEMENT

The authors declare no conflicts of interest.
